# Regional Neural Response Differences in the Determination of Faces or Houses Positioned in a Wide Visual Field

**DOI:** 10.1371/journal.pone.0072728

**Published:** 2013-08-21

**Authors:** Bin Wang, Tianyi Yan, Jinglong Wu, Kewei Chen, Satoshi Imajyo, Seiichiro Ohno, Susumu Kanazawa

**Affiliations:** 1 School of Life Science, Beijing Institute of Technology, Beijing, China; 2 Graduate School of Natural Science and Technology, Okayama University, Okayama, Japan; 3 Computational Image Analysis Program, Banner Alzheimer Institute and Banner Good Samaritan PET Center, Phoenix, Arizona, United States of America; 4 Department of Radiology, Okayama University Hospital, Okayama University, Okayama, Japan; 5 Graduate School of Medicine, Dentistry, Pharmaceutical Sciences, Okayama University, Okayama, Japan; National Institute of Mental Health, United States of America

## Abstract

In human visual cortex, the primary visual cortex (V1) is considered to be essential for visual information processing; the fusiform face area (FFA) and parahippocampal place area (PPA) are considered as face-selective region and places-selective region, respectively. Recently, a functional magnetic resonance imaging (fMRI) study showed that the neural activity ratios between V1 and FFA were constant as eccentricities increasing in central visual field. However, in wide visual field, the neural activity relationships between V1 and FFA or V1 and PPA are still unclear. In this work, using fMRI and wide-view present system, we tried to address this issue by measuring neural activities in V1, FFA and PPA for the images of faces and houses aligning in 4 eccentricities and 4 meridians. Then, we further calculated ratio relative to V1 (RRV1) as comparing the neural responses amplitudes in FFA or PPA with those in V1. We found V1, FFA, and PPA showed significant different neural activities to faces and houses in 3 dimensions of eccentricity, meridian, and region. Most importantly, the RRV1s in FFA and PPA also exhibited significant differences in 3 dimensions. In the dimension of eccentricity, both FFA and PPA showed smaller RRV1s at central position than those at peripheral positions. In meridian dimension, both FFA and PPA showed larger RRV1s at upper vertical positions than those at lower vertical positions. In the dimension of region, FFA had larger RRV1s than PPA. We proposed that these differential RRV1s indicated FFA and PPA might have different processing strategies for encoding the wide field visual information from V1. These different processing strategies might depend on the retinal position at which faces or houses are typically observed in daily life. We posited a role of experience in shaping the information processing strategies in the ventral visual cortex.

## Introduction

The human visual cortex is organized hierarchically. The visual information from retinal ganglion cells is eventually processed in the visual cortex. In the hierarchy of visual cortical areas, the primary visual cortex (V1) is essential for visual information processing, as most or all of the input to the higher cortical areas passes through V1. A number of strategies are used for efficient information processing within this hierarchy, including linear and nonlinear filtering [1], [2]. These strategies are used with the aim of creating different compact visual and functional representations in the organization of the visual cortex [3]–[6].

The human visual system is divided into central and peripheral vision [7]. The visual system seems to represent central stimuli with a fair degree of fidelity, but it more crudely encodes stimuli in peripheral field. Even with such imprecise encoding, the visual stimuli from our peripheral vision are nonetheless important in determining eye movements [8], [9] and in object-motion perception [10], for example. Understanding the information available to the visual system in the peripheral visual field is the key to understanding our visual capabilities and limitations. Despite the importance of peripheral vision, there is little understanding of the information available to the visual system and of visual representation. Peripheral vision has mostly been characterized in terms of the reductions in resolution or contrast sensitivity as the eccentricity increasing [11], [12].

In the hierarchy of visual cortical areas, the ventral and lateral occipital-temporal cortex is responsible for the high-level visual object processing [5], [13]–[15]. Multiple cortical regions are characterized by their consistent preferential response to specific visual categories, such as faces (fusiform face areas, FFA) [16], houses and places (parahippocampal place area, PPA) [17], [18], words (visual word form areas, VWFA) [19], [20] and objects (lateral occipital complex, LOC) [21].

Central-peripheral organization of the category-selective areas is discovered in the human visual cortex [22], [23]. In the ventral visual cortex, the lateral regions, such as FFA and VWFA, represent foveal eccentricities and the medial regions, such as PPA, represent peripheral eccentricities [22], [23]. Since the discovery of central-peripheral organization, more differences have been found in these object-selective areas. For instance, FFA and LOC show a greater magnitude neural responses to lower field images than to upper field images [24]–[26], whereas PPA shows a significantly greater magnitude neural responses to upper field images than to lower field images [25], [27]. These differences in the high-order, category-selective areas imply uniform processing of objects at these different positions. However, Yue and his colleagues assumed that FFA only had a neural response to the local contrast corrected by the function of V1 and did not include any additional face-selective components in central visual field. They stated that the neural activity ratios between V1 and FFA were constant as the eccentricities increasing in central visual field [26]. However, in wide visual field, the neural activity relationships between V1 and the ventral category-selective areas (FFA and PPA) are still unclear.

**Figure 1:**
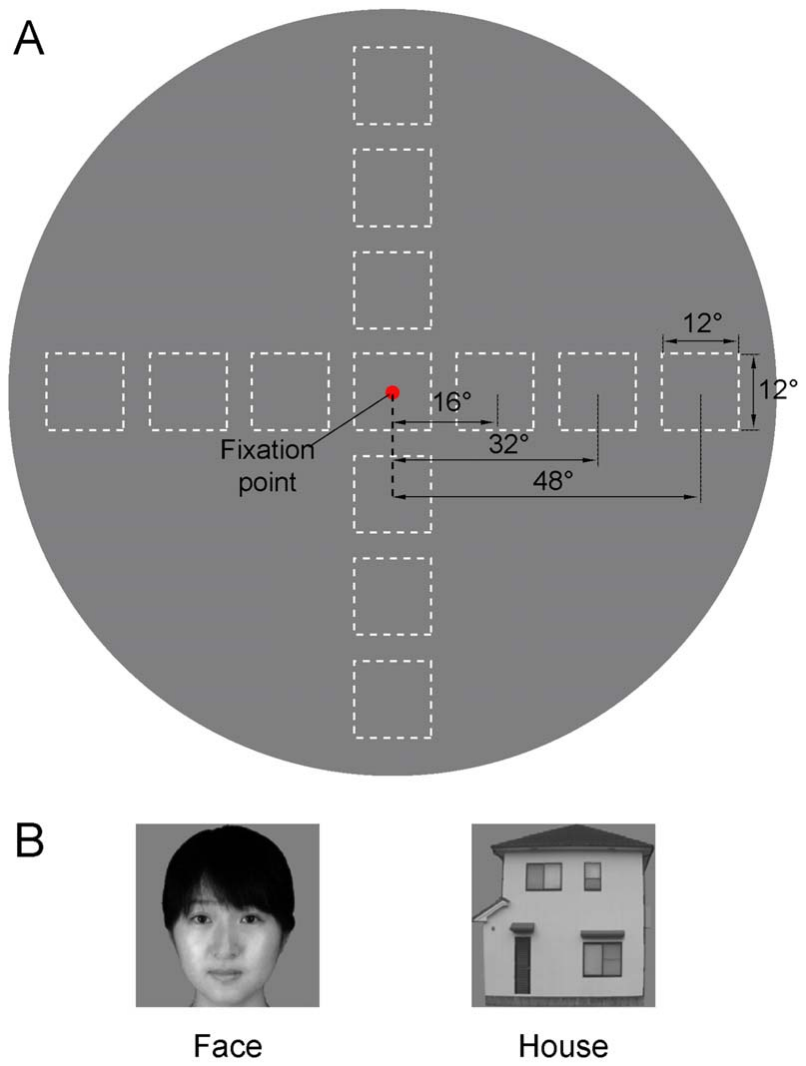
Stimulus configuration and example of stimulus images. (A) The images were either centered at the fixation point (0°) or centered at 16°, 32° or 48° for the left horizontal, right horizontal, upper vertical, or lower vertical meridians. The squares with the dashed lines indicate the stimulus positions in the spherical screen. The red dot indicates the fixation point. Within a given block, images of faces or houses were presented in one of the visual field positions. (B) Example images of a face and a house are shown. The images of the faces shown here do not depict the actual stimuli and are intended only as examples. We have received written permission (as outlined in the PLoS consent form) to use the photograph for the illustration of the stimuli in the publications.

Here, we tried to address this issue by using functional magnetic resonance imaging (fMRI) and wide-view presentation system with up to 60° of eccentricity [14], [28]. The subjects were presented with a face and a house, both of which were centrally located along the left horizontal, right horizontal, upper vertical and lower vertical meridians, arranged in 4 levels of eccentricities (0°, 16°, 32°, 48°) at each meridian (Figure 1). V1 and the ventral category-selective areas (FFA and PPA) had different trends of decreased neural activity as the eccentricities of the images of the faces and houses increased. This study demonstrated that FFA and PPA had differences ratio relative to V1 (RRV1) for their neural response amplitudes in 3 dimensions. The differential RRV1s could be viewed as a processing strategy for encoding the images of the faces and houses with variations in eccentricity and meridian.

## Materials and Methods

### Subjects

MR imaging was performed at the Hospital of Okayama University. Eight subjects (6 males, 2 females), aged 22–25 years with a mean age of 23 years, participated in the study. All of the subjects were right-handed and had normal vision. Data from only 7 of the 8 subjects were included in the following analyses because one subject exhibited significant head movements during the scan. The experiments were performed with the written consent of each subject and were approved by the Ethics Committee of Okayama University Hospital.

### Presentation of Stimuli

The stimuli were projected on a wide-view visual presentation system, which had been upgraded from a previous version [28], [29]. The subjects viewed the stimuli on a hemisphere 52 mm in diameter; the curvature radius of this hemisphere was 30 mm. The mean distance between the subjects' eyes and the screen was 30 mm. The subjects wore contact lenses to focus on the stimulus, and the visual field of stimulus was 120° horizontal × 120° vertical, or 60° of eccentricity.

### Position Experiments

The position experiments utilized grayscale images of human faces and houses. The face images were taken from the FEI face database (http://fei.edu.br/~cet/facedatabase.html), and the houses images were photos taken in Okayama City. The objects were presented at a variety of positions and grayscale backgrounds (Figure 1B). The position experiments utilized 48 unique images from each category. The images subtended a 12° visual angle at each position. We chose to use a constant image size because the magnification factors in the face- and house-selected areas were unknown, and the magnifications at the center and periphery were quite different. We wished to compare the neural activation corresponding to the images of the faces and houses at different positions throughout the central and peripheral visual fields. The images were centered at the fixation point (0° eccentricity) and were centered at 16°, 32° and 48° of eccentricities along 4 meridians: the left horizontal meridian, right horizontal meridian, upper vertical meridian and lower vertical meridian. A total of 13 positions were arranged in the 4 levels of eccentricities (0°, 16°, 32° and 48°) for each meridian (Figure 1A).

The position experiments included 6 runs of block design experiment. Each run contained one 8-s block for each position and category combination; thus, the session contained 26 blocks per run (2 categories × 13 positions). The image blocks were interleaved with 8-s baseline blocks (a grayscale screen with a central fixation point). In each image block, a series of images from one category (face or house) were shown at a specific position in random order. The images were shown at a rate of 1 Hz (800 ms per image, with a 200 ms inter-stimulus interval). During the scanning process, the subjects were instructed to categorize each image while fixating on a red point (a red disk 1.8° in diameter that was present for the duration of the experiment). When the red disk dimmed, the subjects reported their categorization with two buttons that corresponded to either a face or a house. The dimming prompts lasted 1.2 s, with a 1.8- or 3.8-s interval between the prompts, and it was not synchronized with the stimulus onsets. The button presses that occurred outside of the 1.2-s period following a prompt were ignored. The fixation task was primarily used to ensure that the subjects maintained their fixation during the scans. Before scanning, the subjects practiced this task to minimize false alarms and to maintain their focus on the fixation point. Behavioral responses were collected during the scanning using a magnet-compatible button box connected to the stimulus computer.

### Retinotopic Mapping Experiments

To identify the retinotopic areas of the visual cortex, the clockwise rotating wedge and expanding ring stimuli were employed [28], [30]–[32]. These stimulus apertures contained 100% contrast black-and-white checkerboard patterns, and they phase-reversed at a temporal frequency of 8 Hz at an eccentricity ranging from 2.4° to 60°. The wedge stimulus with boundaries of 22.5° was slowly rotated clockwise around a red fixation disk (approximately 1°) presented at the center of the stimulus. The wedge rotated at 22.5° steps, remaining at each position for 6 s before moving to the next position. The eccentricity of the expanding rings ranged from 2.4° to 60°, and the width of the ring stimuli was expanded in exponential increments. The corresponding ring sizes were 1.2°, 1.8°, 2.7°, 4.0°, 6.0°, 9.0°, 13.4° and 20.0°. These expanding ring stimuli were moved in 8 discrete steps and remained at each position for 6 s before automatically expanding to the next position. All of the experiments involved passive viewing, and the subjects were required to maintain their gaze on the red fixation disk in the center of the screen that flickered at a temporal frequency of 4 Hz throughout the scan. Six complete cycles of rotations and checkerboard expansions were conducted.

### Image Acquisition

The imaging was performed using a 3-Tesla MR scanner (Siemens Allegra, Erlangen, Germany). For the functional series, we continuously acquired images with 30 slices using a standard T2-weighted echo-planar imaging (EPI) sequence (TR = 2 s; TE = 35 ms; flip angle = 85°; 64×64 matrices; in-plane resolution: 2.3×2.3 mm; slice thickness: 2 mm with a gap of 0.3 mm). The slices were manually aligned approximately perpendicular to the calcarine sulcus to cover most of the occipital, posterior parietal and posterior temporal cortices. After the functional scans, high-resolution, sagittal, T1-weighted images were acquired using a magnetization-prepared rapid gradient echo sequence (MP-RAGE; TR = 1800 ms; TE = 2.3 ms; matrix 256×256×224; 1-mm isotropic voxel size) to obtain a 3D structural scan.

### Data Preprocessing

The anatomical and functional images were analyzed using the BrainVoyager QX 2.11 (Brain Innovation, Maastricht, The Netherlands). The anatomical images were segmented for the identification of the white/gray matter boundaries and were then used for cortical surface reconstruction and inflation [33]–[35]. In each functional run, the first 2 volumes were discarded to ensure that the steady state had been reached. The functional data were preprocessed with motion and scan time corrections and high-pass temporal filtering (0.01 Hz) before statistical analysis [33]. Spatial smoothing, using a full-width, half-maximum Gaussian kernel of 4 mm, was applied to the position experiments data but not to the retinotopic mapping data. The functional data were transformed into the conventional Talairach space, yielding a 4D data representation [36].

### General Linear Model

We applied a general linear model (GLM) to the position experiments data on a voxel-by-voxel basis. This boxcar function was convolved with a double-gamma hemodynamic response function to account for the hemodynamic effects [37]. To combine the 6 runs of position experiments for each individual, a second-level analysis was performed using a fixed-effects model to estimate the blood oxygen level-dependent (BOLD) response amplitudes for each stimulus condition using a fixed effects analysis of variance (ANOVA). All statistical analyses used the statistical threshold of p<0.05 with false discovery rate (FDR) correction and a cluster threshold of 20 mm^3^. The response maps were rendered on a cortical surface from a high-resolution structural MRI scan of a standard brain based on the Talairach coordinates.

### Retinotopic Mapping Analysis

Our retinotopic mapping experiments employed a standard phase-encoded retinotopy design [28], [30]–[32]. For the polar angle and eccentricity mapping, the stimulation blocks were modeled by boxcar functions convolved with a double-gamma hemodynamic response function [37]. The stimulus-driven modulation of the BOLD response in each functional voxel was revealed via a linear correlation map analysis. This phase was mapped into physical units by identifying the stimulus parameter (polar angle or eccentricity) corresponding to the time. The color-coded cortical regions were classified based on an r-value threshold of 0.25. To aid in visualization, the retinotopic maps were projected onto computationally flattened representations of the cortical surface.

### Region of Interest Analysis

**Figure 2:**
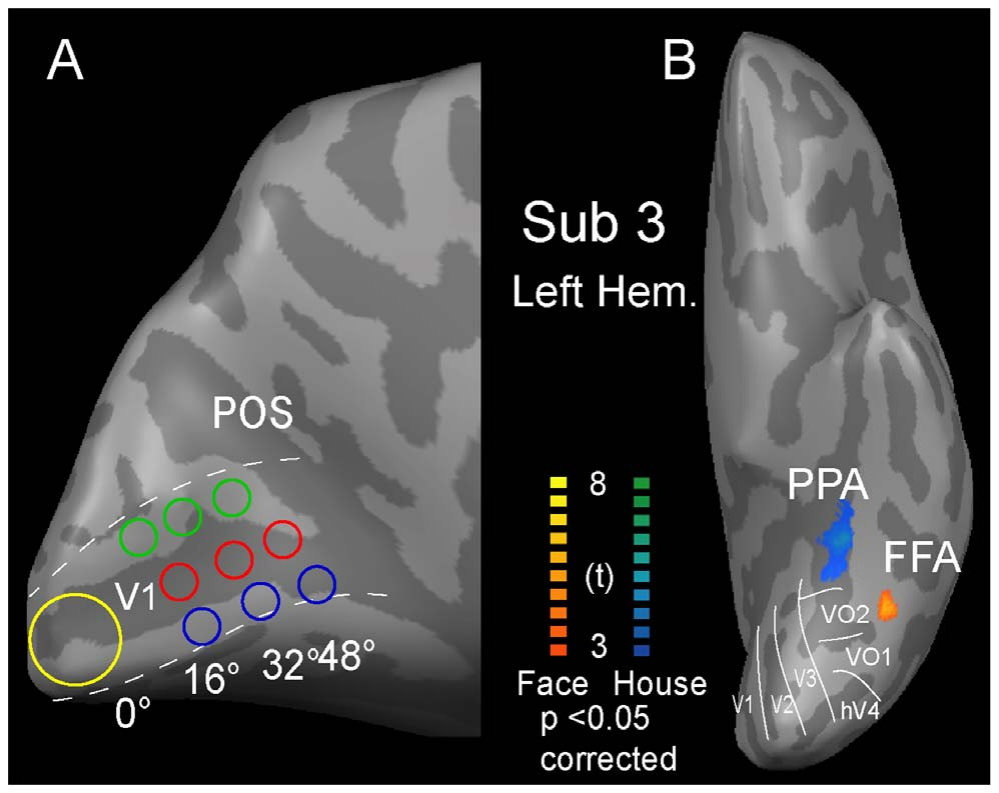
The locations of the ROIs on the visual cortex. (A) A central view of the inflated cortex shows V1 region, shown with dash lines. The 10 ROIs in V1 are indicated by the colored disks. The yellow disk corresponded to the central position. The red disks corresponded to the ROIs of the contralateral horizontal positions, the blue disks corresponded to the ROIs of the upper vertical positions, and the green disks corresponded to ROIs of the lower vertical positions. (B) The ventral view of the inflated cortex shows the locations of FFA and PPA. The face-selective area is shown by the red-yellow color, and the house-selective area is shown by the blue-cyan color.

In V1, as the stimulus positions moved from the fovea to the periphery of the retina, the locations of the response area varied from the posterior to the anterior areas of the calcarine sulcus. The regions of interest (ROIs) were individually defined for each participant based on the position experiments data and V1 mask obtained individually from retinotopic mapping. This method was performed by contrasting all the stimuli at one position with all the other positions using the contrast threshold of p<0.05 corrected with the FDR and a spatial extent of at least 20 mm^3^ (Figure 2A). For the positions of the face and house stimuli (Figure 1), 10 functional ROIs, corresponding to the 4 eccentricities and 3 meridians positions that occupied half of the visual fields, were defined in each hemisphere. The functional ROIs were defined separately for the images of the faces and houses and were referred to as Face-V1 and House-V1. The mean Talairach coordinates, cluster volume, and defined number of each ROI are shown in Table S1. Images faces and houses activated similar V1 extents at all the positions (all: paired t-test p>0.2). The neural activities in response to the images of the faces or houses at each stimulus position were assigned as the BOLD response amplitude in a matched ROI.

Using the position experiments data, FFA and PPA were defined based on the combined activations from all 13 locations. The FFA ROIs were defined as a region that responded more strongly to images of faces than houses [4], [16], [38]; however, the FFA ROIs were identified as a region that responded more strongly to images of houses than faces [4], [17], [39] (Figure 2B). The contrast threshold was p<0.05, the data were corrected for FDR, and the spatial extent was 20 mm^3^. The FFA ROIs were defined in the right hemisphere for all of the subjects and in the left hemisphere for 6 out of the 7 subjects. The PPA ROIs were defined in both hemispheres for all 7 subjects. We extracted the magnitude of the neural responses to the images of faces or houses in FFA or PPA for each position. Then, the statistical analyses were applied linear mixed model for repeated measures by using the SPSS software (version 16.0; SPSS Inc., Chicago, Ill).

### Signal Intensity Mapping

To evaluate the quantities of MRI signal quality in V1 (calcarine sulcus), the signal intensity map (temporal signal-to-noise: the ratio of the average signal intensity to the signal standard deviation) was measured for the EPI data [40]. The signal intensity values for the ROIs defined within V1 (Figure 2A) were also measured.

## Results

### Position Sensitivity in a Wide Field

In the position experiments, behavior performances at each position are listed in Table S2. Some subjects had no or less response to the images of faces or houses at the most peripheral positions, and then resulted in response times with miss values. In the 4 meridians, linear mixed models for repeated measures with factors of eccentricity (0°, 16°, 32°, and 48°) and category (faces or houses, 4 × 2) were applied. Neither the response times nor the accuracy was significantly affected by category, and there was no significant interaction between category and eccentricity (p>0.05). We found a significant effect of eccentricity for the response times only at the lower vertical positions (p = 0.05) and for the accuracy at 4 meridian positions (p<0.01). The detailed statistical values are listed in Table A in File S1. To take the meridian effects into consideration, a linear mixed model for repeated measures with factors of eccentricity (16°, 32°, and 48°), meridian (left horizontal, right horizontal, upper vertical and lower vertical positions) and category (faces and houses, 3×3×2) revealed that both the response times and the accuracy were significantly affected by eccentricity and meridian (p≤0.002), and there was significant interactions between meridian and eccentricity (p≤0.004). The detailed statistical values are listed in Table B in File S1. A pairwise comparison showed that the 48° positions had shorter response times and lower accuracy than the 16° and 32° positions. The right horizontal positions had shorter response times and lower accuracy compared to the other 3 meridians (p ≤ 0.002).

### Neural Activity Maps for Images of Faces and Houses

**Figure 3:**
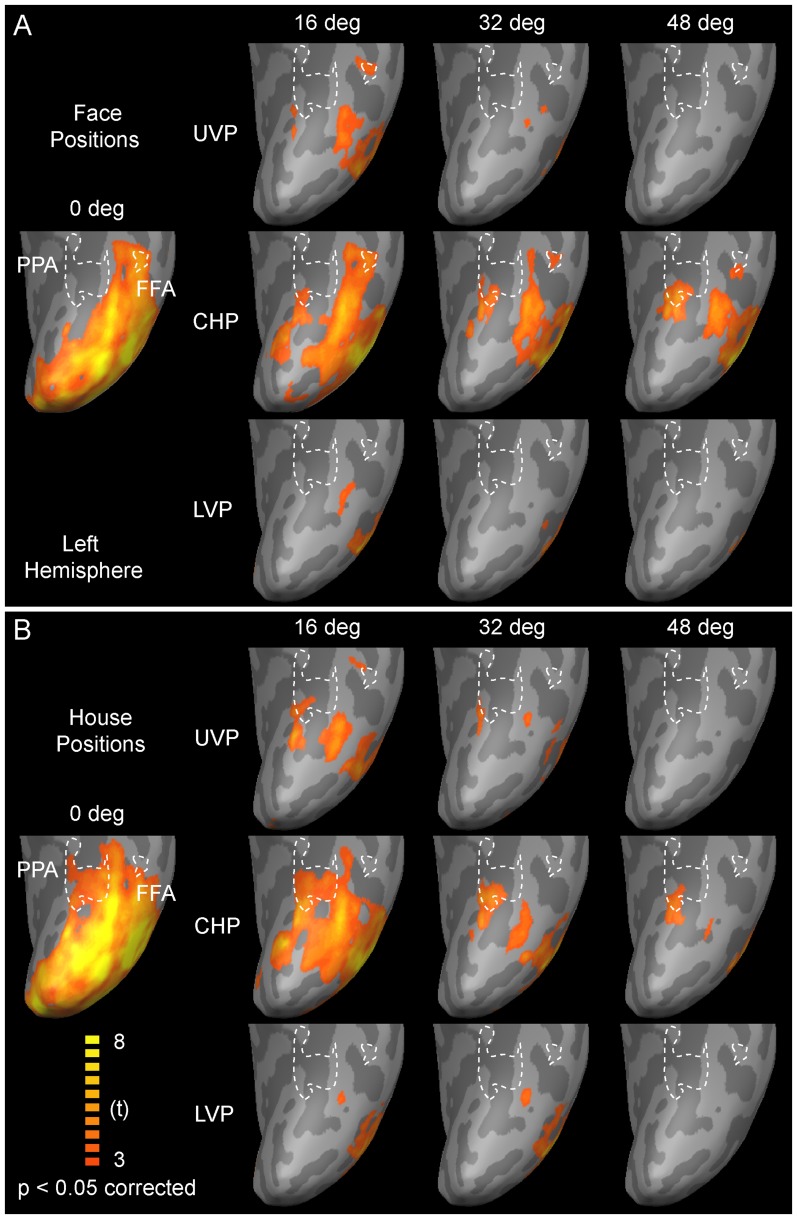
The mean neural responses of 7 subjects to images of faces or houses at the right horizontal, upper vertical, and lower vertical positions in ventral category-selective areas (FFA and PPA). (A) The neural responses to face images. (B) The neural responses to house images. Abbreviations: CHP, contralateral horizontal positions; UVP, upper vertical positions; LVP, lower vertical positions.

In line with the behavior results, the neural activity in the visual cortex also had significant eccentricity and category effects. Figure 3 shows the mean neural activity maps of 7 subjects. We present the statistical maps at a threshold of p<0.05 with FDR correction and a cluster threshold of 20 mm^3^. In the ventral visual cortex, the central positions had stronger neural activities compared with peripheral positions; the contralateral horizontal positions had stronger neural activities compared with the upper vertical positions and the lower vertical positions. The positions along the vertical meridian straddled each hemifield equally, thus, the stimuli sizes along the vertical meridian were about half of those along the horizontal positions, and then stronger neural activities were found at the horizontal positions than the vertical positions. Generally, these results were consistent with the retinotopic organization of visual cortex [28], [30]–[32].

### Mean Response Magnitude

**Figure 4:**
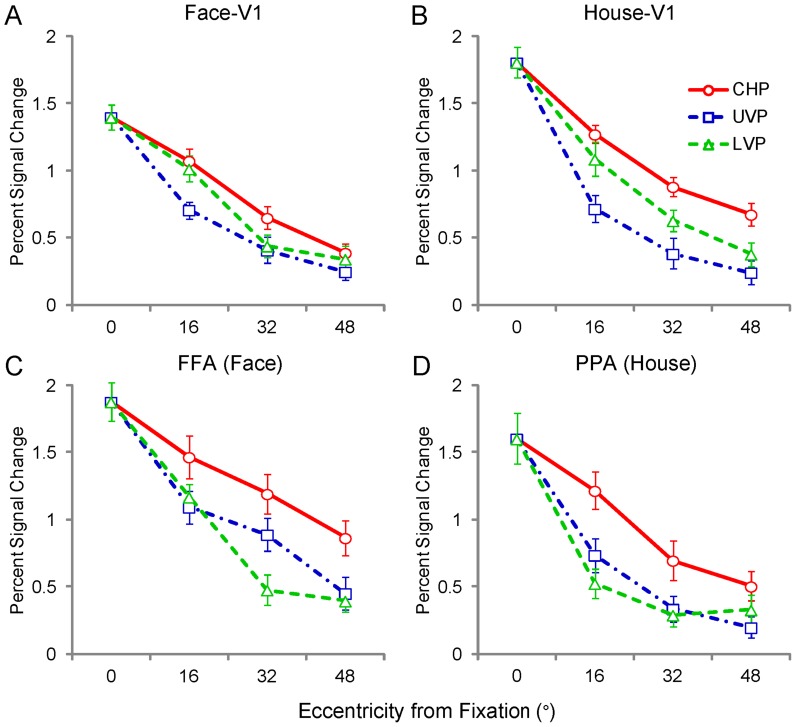
Mean response amplitudes in response to images of faces and houses at each of the 10 stimulus positions in V1 and ventral category-selective areas (FFA and PPA). Generally, the significant neural activities decreased as eccentricity increased in V1 (A, B) and ventral category-selective areas (FFA and PPA, C, D). There were significant effects of eccentricity and region for each meridian positions (all: p ≤ 0.02), except for the upper vertical positions (p = 0.18) in V1. Significant interactions between eccentricity and region were found at the upper and lower vertical positions in V1 and in the upper vertical positions in the ventral category-selective areas (all: p≤0.03). Considering the dimension of meridian, there were significant main effects of meridian in V1 and ventral category-selective areas (FFA and PPA, p<0.001). The abbreviations are the same as those used in Figure 3.

The neural responses in each ROI in bilateral hemispheres are shown in Figure S1. Pooling the results from two hemispheres, we measured the mean magnitude of the response to the 10 positions, considering that one central position and 3 eccentricities in 3 meridians (contralateral horizontal, upper vertical, and lower vertical meridian) occupied half of the visual fields. In V1 and ventral category-selective areas, Face-V1 and FFA were the ROIs defined for the face images and House-V1 and PPA were the ROIs defined for the house images. Figure 4 shows the mean response magnitude in each ROI. Firstly, we considered each meridian separately, including the contralateral horizontal positions, upper vertical positions, and lower vertical positions. In V1, there were some missing values in the neural response magnitude that could not be defined by the contrast in each position (Materials and Methods). Linear mixed models for repeated measures with factors of eccentricity (0°, 16°, 32° or 48°) and region (for faces and houses, 4×2) were applied for V1 areas (Face-V1 and House-V1, Figure 4A, B) and the ventral category-selective areas (FFA and PPA, Figure 4C, D); the statistical values are listed in Table C in File S1. Generally, there were significant main effects of eccentricity in V1 and the ventral category-selective areas (all: p<0.001), which consisted of the behavior result (Table B in File S1) and the neural activity on the visual cortex (Figure 3). In V1, the only significant main effects of region were found at the contralateral horizontal and lower vertical positions (p ≤ 0.02), and significant interactions between eccentricity and region were found at the upper vertical positions and lower vertical positions (p ≤ 0.03), except for V1 at the contralateral horizontal positions (p = 0.21). The faces had weaker neural response magnitudes than the houses at the contralateral horizontal positions, which resulted from the relatively smaller size of the face images compared to the house images. The faces were always shown as an ellipse shape, and the houses were always shown as a square shape. Moreover, the difference between the magnitudes of the neural response to the faces and houses became weaker at the upper vertical positions and lower vertical positions. In the ventral category-selective areas (Figure 4C, D), we also found significant main effect of eccentricity at all three meridians positions (p<0.001), which were similar as in V1. However, FFA had stronger neural response magnitudes than PPA, which contrasted with the results from V1. Significant main effects of region (p ≤ 0.02) were found at all the three meridians positions. A significant interaction between eccentricity and region was found only at the lower vertical positions (p = 0.01).

From the statistical results above, the meridian factor had effects on the neural response magnitude both in V1 (Figure 4A, B) and the ventral category-selective areas (Figure 4C, D). The neural response magnitudes at the peripheral 3 eccentric level positions were applied. Linear mixed models for repeated measures with factors of eccentricity (16°, 32° and 48°), meridian (contralateral horizontal, upper vertical, and lower vertical positions), and region (3 × 3 × 2) were applied; the statistical values are listed in Table D in File S1. Significant main effects of eccentricity, meridian, and region were found in V1 and ventral category-selective areas (all: p ≤ 0.03). There was a significant interaction between meridian and region (p = 0.03) in V1, while in FFA and PPA, there was a significant interaction between eccentricity, meridian, and region (p<0.001). Pairwise comparisons showed that the neural response magnitude at 3 meridian positions were significantly different (p≤0.01).

### Relative to Central Position

**Figure 5:**
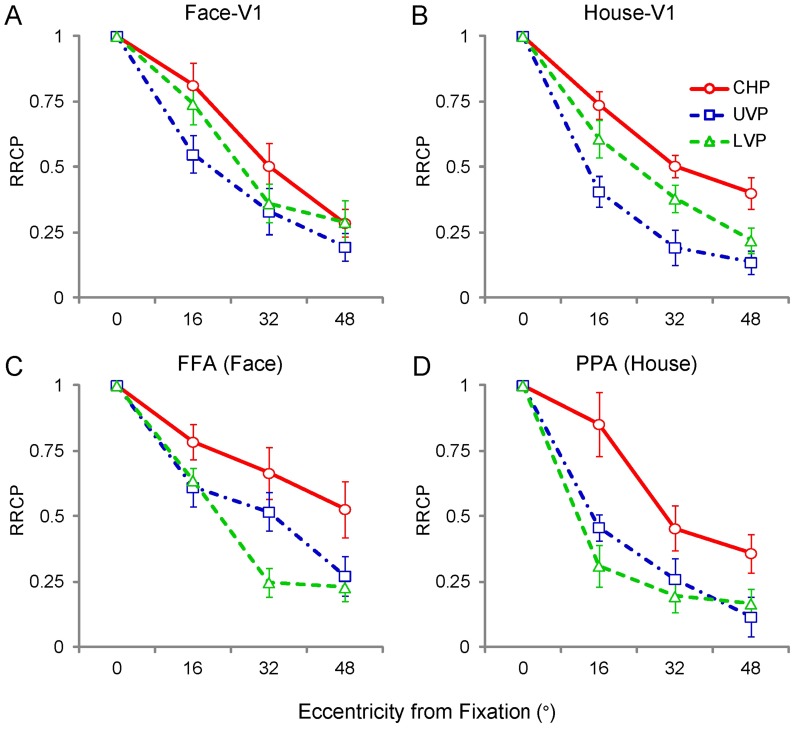
Mean RRCPs in V1 and ventral category-selective areas (FFA and PPA). The central position had an RRCP of 1. In general, similarly to the mean response amplitudes (Figure 4), the RRCPs decreased as the eccentricity increased in V1 (p<0.001, A, B) and ventral category-selective areas (FFA and PPA, C, D). There were significant effects of eccentricity (p<0.001) at each meridian positions and significant effects of region at the upper and lower vertical positions (p≤0.01) in the ventral category-selective areas. Significant interactions between eccentricity and region were found (p = 0.002) only at the contralateral horizontal positions in the ventral category-selective areas. Considering the dimension of meridian, there were significant effects of meridian in V1 and ventral category-selective areas (FFA and PPA, p<0.001). The abbreviations are the same as those used in Figure 3.

We found differences in response magnitude of the neural activities in V1, FFA and PPA. The neural activities in response to the houses were stronger than those in response to the faces in V1, but FFA displayed much greater neural activities than PPA, especially for the central position. We normalized the neural response amplitude by calculating the ratio relative to central position (RRCP) for each position and ROI (RRCP = neural response amplitude at each position / neural response amplitude at central position). A ratio of 1 meant that the neural response amplitude of a position was the same as that of the central position. The mean results of the RRCPs are shown in Figure 5. The values of RRCPs were subjected to the same statistical analysis as the mean response magnitude above. The detailed statistical values are listed in Table E in File S1. In V1 (Figure 5A, B), for each meridian, there were only significant effects of eccentricity (all: p<0.001). Taking the meridian effect into consideration, the significant main effect of eccentricity and meridian were found (all: p<0.001). In addition, no significant interactions were identified among eccentricity, meridian, and region (all: p>0.1). These results indicated that the neural activities in V1 were affected by eccentricity and meridian but not by the category selectivity.

In the ventral category-selective areas (Figure 5C, D), the statistical results were similar to those of the neural responses. For each meridian, there were significant main effects of eccentricity (all: p<0.001) and region at the upper and lower vertical positions (all: p ≤ 0.01). In addition, significant interactions between eccentricity and region were found only at the lower vertical positions (p = 0.002). Taking the meridian effect into consideration, significant main effects of eccentricity, meridian and region were found (all: p ≤ 0.002). There was significant interaction between eccentricity, meridian and region (p = 0.02). The detailed statistical values are listed in Table F in File S1. These results indicated that the neural activities in the ventral category-selective areas were affected by meridian and region, in addition to eccentricity.

### Relative to V1

**Figure 6:**
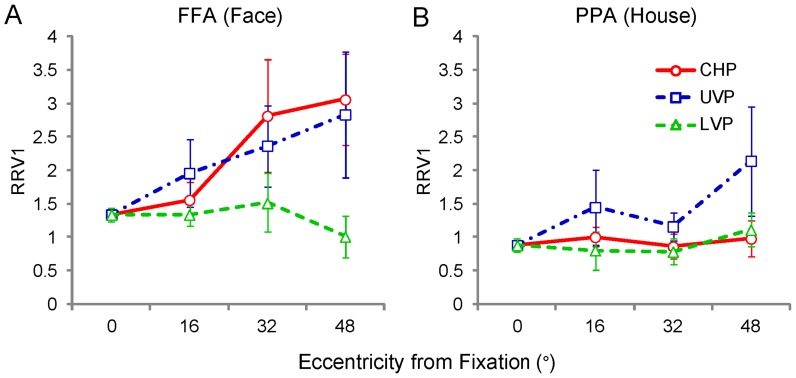
RRV1s in the ventral category-selective areas for images of faces and houses. (A) RRV1s in FFA for images of faces, and (B) RRV1s in PPA for images of houses. There were significant effects of region (p ≤ 0.05) for all 3 meridians and main effects of eccentricity at the contralateral horizontal and upper vertical positions (p ≤ 0.03). Moreover, considering the dimensions of meridian, there was a significant effect of meridian and region (p<0.001). Significant interactions between meridian and region were found (p = 0.005). The abbreviations are the same as those used in Figure 3.

The results of the mean response amplitudes and RRCPs implied that the neural activities to the faces and the houses in V1 had a consistent effect of eccentricity and meridian. In contrast, there was a greater difference on the effect of eccentricity and meridian between FFA and PPA. We proposed that the neural representations of the images of faces and houses within a wide field included differences in V1 and ventral category-selective areas. To determine the conversion from V1 to FFA and PPA, we calculated the ratio relative to V1 (RRV1) for the neural response amplitude of each position and ROI (RRV1 = neural response amplitude in FFA or PPA / neural response amplitude in V1). When the neural response amplitude in FFA or PPA was greater than that in V1, the RRV1 was greater than 1, and when the amplitude was smaller, the RRV1 was smaller than 1. Only the positive response amplitudes were used for the final calculations. Figure 6 shows the mean RRV1s in each position for each ROI; the statistical values are listed in Table G in File S1. Firstly, linear mixed models for repeated measures with factors of eccentricity and region (4 × 2) revealed main effects of eccentricity at the contralateral horizontal positions and upper vertical positions (p ≤ 0.03). An interaction between eccentricity and region was found only at the contralateral horizontal positions (p = 0.03). Pairwise comparisons of eccentricity reveal a different effect of eccentricity in each meridian positions and ROIs. At the contralateral horizontal positions, the 32° and 48° positions had bigger RRV1s than the 0° and 16° positions in FFA (p ≤ 0.05). Along the upper vertical median, the 32° and 48° positions had bigger RRV1s than the 0° positions in FFA, and the 48° positions had bigger RRV1s than the 0° positions in PPA (p<0.05). In addition, there were main effects of region at the contralateral horizontal, upper vertical and lower vertical positions (p ≤ 0.05). The larger RRV1s in FFA than PPA were consisted with the results of mean response amplitudes; the faces elicited weaker neural activities in V1 area and stronger neural activities in the category-selective areas, comparing with house.

These two-factor statistical analysis results revealed that the RRV1s also had different effects of meridian in FFA and PPA (Figure 6). To analyze the meridian effect, a linear mixed model for repeated measures with factors of eccentricity, meridian and region (3 × 3 × 2) revealed a main effect of meridian and region (p<0.001) and an interaction between meridian and region (p = 0.005). The statistical values are listed in Table H in File S1. In FFA, a pairwise comparison showed that the contralateral horizontal positions and upper vertical positions had a greater RRV1 than the lower vertical positions (p<0.001), and the contralateral horizontal positions caused no differences between the upper vertical positions (p = 0.8), while in PPA, the upper vertical positions had a greater RRV1 than the contralateral horizontal positions and the lower vertical positions (p ≤ 0.04), and the contralateral horizontal positions caused no difference between the lower vertical positions (p = 0.9).

### Neural Response to the Checkerboards Rings

**Figure 7:**
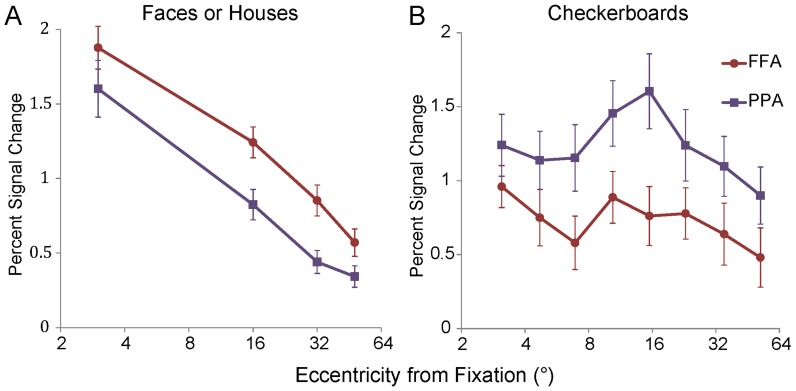
Mean magnitudes of neural response to the images of faces (houses) and the checkerboard rings. (A) The combined magnitudes of neural response to the images of faces (houses) at the 3 meridian positions in FFA (PPA). The mean response magnitudes of the 3 meridians were consistent with the result described before (Figure 4). (B) The neural response magnitudes to the checkerboard rings in FFA and PPA. It is revealed that a significant effect of eccentricity and region (p = 0.002) and an interaction between eccentricity and region (p = 0.04). The two decreasing sections of the neural response magnitudes were the central 3 rings, which covered the visual field of 8° eccentricities (p = 0.02), and the peripheral 5 rings, which covered the visual field of 8–60° eccentricities (p = 0.004). A significant interaction was found between region and eccentricity for the central 3 rings (p = 0.03). The x-axis of eccentricity is labeled on a base 2 logarithmic scale.

We also measured the neural response magnitudes to the expending of checkerboard rings in the retinotopic mapping experiments (Materials and Methods). In Figure 7, we showed the mean magnitudes of the neural responses to the images of faces (houses) combined across all 3 meridians (Figure 7A) and the response magnitudes to the checkerboard rings (Figure 7B) in FFA (PPA). Linear mixed models with repeated measure of eccentricity and region (FFA and PPA) were applied, and the statistical values are listed in Table I in File S1. There were significant effects of eccentricity and region for both the images of faces (houses) and the checkerboards ring (all: p ≤ 0.01). The checkerboard had significantly stronger neural response magnitudes in PPA than FFA for all the 8 rings (p = 0.002), which was contrary to the neural responses to the images of faces and houses. As the widths of the rings were applied in exponential increments, the response magnitudes had peaks at the fourth or fifth rings, which mainly occupied the eccentricities of 8–18°. Then, there were two decreasing sections of the neural response magnitudes. One section was the central 3 rings, which covered the visual field of 8° eccentricities (p = 0.02), and the other one section was the peripheral 5 rings, which covered the visual field of 8–60° eccentricities (p = 0.004). An interaction between eccentricity and region was found between region and eccentricity for the central 3 rings (p = 0.03), but no interaction was found between region and eccentricity for the peripheral 5 rings (p = 0.12). In addition, there was no interaction between eccentricity and region on the response magnitudes to the faces and the houses (p = 0.42), which was consistent with the peripheral checkerboards.

### Signal Intensity in V1

**Figure 8:**
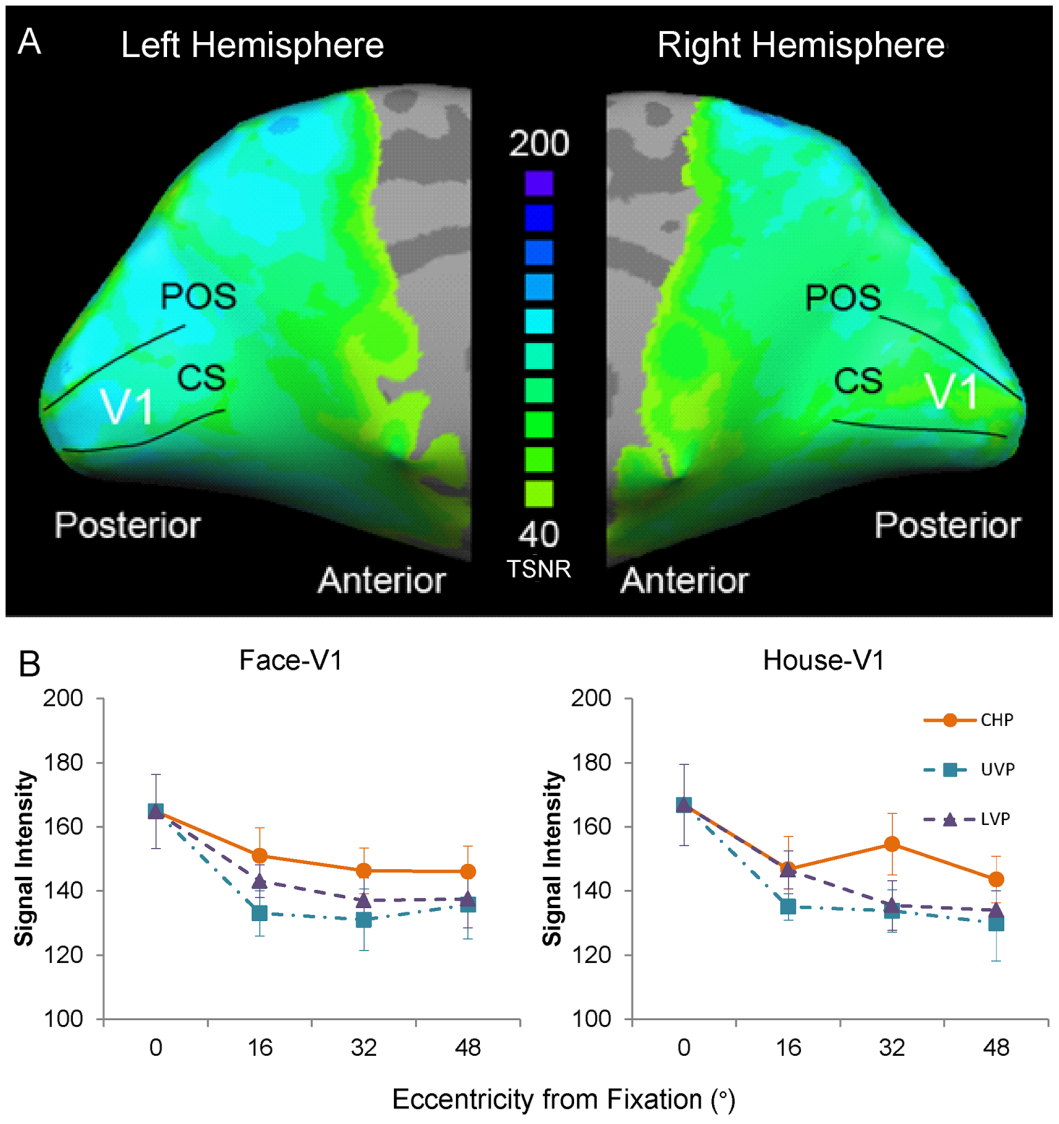
Signal intensity of EPI. (A) Signal intensity maps showing EPI image quality in the calcarine sulcus. The color gradient indicates the mean signal intensity of the smoothed EPI time course data overlaid on the inflated cortex of the Talairach brain. The threshold of the color map was established at a TSNR of 40, and all the blue areas indicate a TSNR of at least 200. Simulations indicate that a TSNR of 40 (indicated in the map by light green) is the minimum necessary to reliably detect the effects between the conditions in the fMRI data [40]. (B) The signal intensities in the ROIs of V1. At the contralateral horizontal positions, the signal intensities in the 4 ROIs (0°, 16°, 32°, and 48°) were not significantly different (p = 0.06). At the upper and lower vertical positions, the signal intensities in the 3 ROIs (16°, 32°, and 48°) were not significantly different (p ≥ 0.35), but they were significant smaller than the signal intensities at the contralateral horizontal positions (p<0.05). The abbreviations are the same as those used in Figure 3.

As demonstrated by the mean signal intensity mapping (temporal signal-to-noise: the ratio of the average signal intensity to the signal standard deviation) of the 7 subjects, the signal quality in the calcarine sulcus was very good, even in the anterior regions of calcarine sulcus (Figure 8A). The simulations indicated that a TSNR of 40 (indicated in the map by light green) was the minimum to reliably detect the effects between the conditions in the EPI data [40]. Note that virtually all of the calcarine sulcus far exceeds this threshold, with many exceeding a TSNR of 200. The signal intensities in the ROIs for the Face-V1 and the House-V1 were also reported (Figure 8B). Using linear mixed models for repeated measures, we found no differences between the ROIs of Face-V1 and ROIs of House-V1 (all: p ≥ 0.86); the statistical values for signal intensity are listed in Table J in File S1. In the ROIs along the contralateral horizontal median, the signal intensities of the 4 eccentric positions (0°, 16°, 32°, and 48°) had no significant effect of eccentricity (p = 0.06). In the ROIs along the upper and lower vertical median, the signal intensities of the 3 eccentric positions (16°, 32°, and 48°) had no significant effect of eccentricity (p ≥ 0.35). We found a significant effect of meridian on the signal intensities (p = 0.03, Table K in File S1). Through pairwise comparison, the signal intensities in the upper and lower vertical median ROIs were no significant differences (p = 0.5) but significantly smaller than those in the contralateral horizontal positions ROIs (both: p<0.05). The ROIs of the contralateral horizontal and central positions were mainly in the calcarine sulcus. However, the ROIs of the upper (lower) vertical positions were located at the gyrus ventral (dorsal) calcarine sulcus. The anatomical difference resulted in different signal intensities in the ROIs at the contralateral horizontal positions and vertical positions. Thus the results of signal intensity verified that the signal quality was not significantly different between the anterior and posterior regions of the calcarine sulcus.

## Discussion

Our study provides a broad-based survey of position information in FFA and PPA located in the ventral visual cortex. We measured the mean response amplitudes to 13 positions in a wide field and then calculated the values of the RRCPs and RRV1s. Important new findings were revealed concerning the different neural processing strategies in the dimensions of eccentricity, meridian and region.

### Different Processing Strategies in the Dimension of Eccentricity

Human vision is divided into central and peripheral vision [7]. Peripheral vision has mostly been characterized in terms of the reductions in resolution or contrast sensitivity as eccentricity increases [41]–[43]. The ability of humans to detect movement is better in peripheral vision than foveal vision, but color discrimination is markedly worse [44], [45]. In the behavior data, lower accuracy was found at the peripheral positions. Consistent with the behavior performance, the mean response magnitudes in V1, FFA and PPA (Figure 4, 5) decreased as the visual stimuli (images of houses and faces) were presented at progressively greater distances from the center of the visual field. The neural response to the checkerboard rings also exhibited a decreasing trend as the eccentricities increased; the central 3 rings covered the visual field of 8° eccentricities, and the peripheral 5 rings covered the visual field of 8°–60° eccentricities (Figure 7B). These results confirmed the central-peripheral organization in the primate visual cortex. After normalizing the neural response magnitudes by dividing the response magnitudes at the central position, the RRCP values decreased with the same trend in V1 and with different trends in ventral category-selective areas (FFA and PPA, Figure 5).

An important new finding was revealed by comparing the RRV1s. As shown in Figure 6, we found that the RRV1s in FFA and PPA had a significant effect of eccentricity (p ≤ 0.03) at the contralateral horizontal positions and upper vertical positions. Furthermore, the differences in eccentricity were mainly found at the contralateral horizontal and upper vertical positions for FFA and at the upper vertical positions for PPA. Measuring the signal intensity in V1 showed that the signal quality in the calcarine sulcus was very good. The signal intensities had no difference between the anterior and posterior regions of the calcarine sulcus (Figure 8). The consistent signal qualities confirmed that the differences in RRV1s were indeed the neural processing difference of the visual cortex but were not induced by dropping the signal quality. These results demonstrated that FFA and PPA had systematic neural variances from the central field to the peripheral field because the RRV1s increased with eccentricity, especially FFA. In the visual cortical areas, V1 is essential for visual information processing. A number of strategies, including linear and nonlinear filtering, are used for efficient information processing in the higher level areas [2]. From our findings, we considered that FFA and PPA had different strategies in the dimension of eccentricity were adopted to process the information from V1, especially FFA.

**Figure 9:**
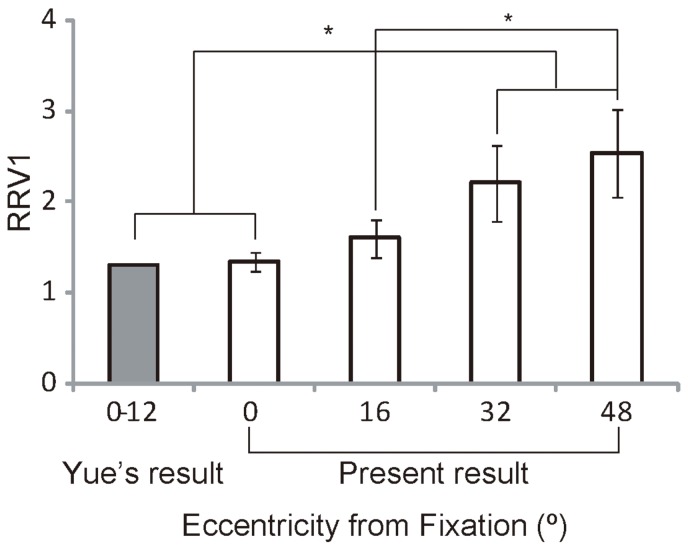
Comparison of our RRV1 results to the results obtained by Yue and colleagues. The stimuli in Yue's study covered the central visual fields, approximately 12° of eccentricity. Our RRV1 results from FFA combined across all 3 meridians were compared to those of Yue. His results agreed with our RRV1 at 0° and 16° of eccentricity, and there was a significant difference with RRV1s at 32° and 48°. The asterisks denote significance (p<0.05).

In contrast, Yue and his colleagues reported that FFA produces neural activities that fit well with the model based on V1 function [26]. They analyzed neural responses along 4 meridians, including the ipsilateral horizontal positions. Their results suggest that the RRV1s of FFA in central visual field are a constant, which was approximately 1.3. In our study, within a wide visual field, the ipsilateral neural responses were weak or negative in V1 and PPA due to the contralateral main neural activities in the human visual cortex in response to stimuli, especially with regard to V1 [26], [46]. The RRV1s were not well suited to the ipsilateral neural responses, thus the ipsilateral neural responses were ignored. Combining all 3 meridians, our RRV1 results from FFA were compared with the results of Yue et al. (Figure 9). At the 0° and 16° positions, the RRV1s of FFA were consistent with Yue's results (t-test, p≥0.16), while in the peripheral positions (32° and 48°), our values were greater than those of Yue (t-test, p≤0.03). Additionally, in our study, the RRV1s at the 32° and 48° positions were significantly greater than those at the central position (p ≤ 0.04), and the RRV1 at the 48° position was significantly greater than those at the 16 ° position. The stimuli in Yue's study covered the central visual fields, with approximately 12° of eccentricity. Obviously, the results from the central visual field did not represent the entire visual field. In combination with the report of Yue [26], it was confirmed that the neural activation in FFA adopted different processing strategies in the dimension of eccentricity, compared to the neural activation function of V1, which showed smaller RRV1s at the central positions and larger RRV1s at the peripheral positions. The human retina has much weaker visual information processing capabilities in the peripheral visual field than the central visual field [47], [48]. Remarkably, the greater RRV1s at the peripheral positions might reflect a compensation mechanism for the peripheral field on the higher visual cortex. In PPA, the 48° positions had greater RRV1s than the 0° positions only in the upper vertical meridian. We considered that the compensation mechanism for the peripheral field was weak in PPA.

### Meridian Difference in Neural Processing

Previous studies demonstrated significant effects of meridian in the higher-level, category-selective areas. PPA showed a significantly greater response magnitude to the upper field images compared to the lower field images. In contrast, the FFA, EBA and LO exhibited opposite effects and greater response magnitudes to the lower field images compared with the upper field images [25], [49]–[51]. In our study, the visual stimuli expanded approximately 54° of eccentricity and FFA and PPA exhibited greater neural activities and the RRCP values at the upper vertical positions than those at the lower vertical positions (p = 0.01). We considered that the result from the wide-field stimuli more accurately reflected this meridian bias.

There were stronger neural responses or RRCPs to the lower vertical positions compared to the upper vertical positions in V1 (Figure 4, 5), which were consistent with the previous reports of V1 [28], [52], [53] and were caused by the larger retinal ganglion density in the lower meridian [47], [48]. Generally, perception at the lower visual field is also superior to that in the upper visual field [54]–[56]. However, lower-biased neural responses were not found at the higher category-selective area, and the upper vertical positions had greater neural activities and RRCP values than the lower vertical positions. The RRV1 values at the upper vertical positions were also larger than those at the lower vertical positions (p ≤ 0.04, Figure 6). We inferred that the larger RRV1s in the upper vertical meridian might comprise a compensation mechanism for the lower vertical meridian biased retinal ganglion density and V1 neural activities. Moreover, in our study, the behavior performance was not different between the upper and lower vertical positions, which also supported this compensation mechanism.

Thus, according to this processing strategy model of a compensation mechanism based on meridian, approximately equal neural activities were observed for images of faces and houses at both the upper and lower vertical positions. Moreover, previous studies have reported lower biases in the FFA, EBA and LO [25], [49]–[51], which might be caused by the intense lower bias in V1. The upper biases in the PPA in the mean response amplitude results [25], [49]–[51] were justified, as the much stronger upper biases compensated for the lower biases in V1.

### Difference between FFA and PPA

In present study, within wide visual fields of 60° eccentricities, the subject had no difference on the response time and accuracy for the face and house images. As the result, the images of both faces and houses elicited different neural responses magnitude for each position in V1 and ventral category-selective areas (FFA and PPA, Figure 4). Face images had significant smaller magnitude responses than house images in V1, and neural responses to face images in FFA were significantly greater than the neural responses to house images in PPA. We found smaller neural activities to checkerboards in FFA than that in PPA. It was implied that FFA and PPA had different strategies for processing images of faces and houses. Furthermore, the RRCPs had only significant difference in ventral category-selective areas (FFA and PPA), but not in V1 (Figure 5). The RRV1s result also confirmed a difference between FFA and PPA (Figure 6). In FFA, face images generated much greater RRV1s than house images in PPA (p ≤ 0.05), especially in the contralateral horizontal meridian positions. These results demonstrate that different processing strategies were also employed in FFA and PPA. From the present results, greater RRV1s indicate that the neural responses had greater ratios added to V1 functions. In FFA, the RRV1 values at all positions were greater than 1 (t test, p<0.0001), which means that FFA had an amplifying effect. However, in PPA, the RRV1 values at all the positions had no significant difference between value of 1 (t test, p = 0.37). In another word, there was no amplifying effect in PPA to processing house images. These results demonstrated that the larger RRV1s were associated with the central representation in FFA, and the smaller RRV1s were associated with the peripheral representation in PPA. The differences of RRV1s reflected more information about the different neural function between FFA and PPA, in addition to previous reports of the central-peripheral organization of the human category-selective areas [22], [23], [25], [57]. We hypothesized that the different neural processing strategies existed between face image processing in FFA and house image processing in PPA.

According to the central-peripheral organization for category-selective areas [22], [23], FFA is associated with center-biased representations in the cortex and PPA is associated with periphery-biased representations in the cortex [22], [23], [25], [57]. FFA had a stronger neural response to stimuli in the central fields than the peripheral fields, and PPA had stronger neural responses to stimuli in the periphery than the central fields. However, in the present results, we did not find the central-peripheral bias of neural activities in FFA and PPA. The neural response magnitudes and the RRCPs had similar decay trends at the contralateral horizontal and upper vertical positions. These differences between the present result and previous reports were clear in the comparison of the neural response to checkerboards. There as a significant interaction between region and eccentricity for the central 3 eccentric rings, covering the visual field of 8° eccentricities (p = 0.03), which meant that the FFA had a steeper decreasing trend compared to PPA. Within more peripheral fields, no interactions between region and eccentricity were found. The stimuli used in previous reports mainly covered visual fields with an eccentricity of 10°. The characteristics of the central-peripheral organization are limited [22], [23], [25]. In the present study, the stimuli covered a visual field with an eccentricity of 60°, and neural responses to images of faces, houses, and checkerboards had trend of decreasing as the eccentricities increased, but these data also exhibited differences (Figure 7).

At the contralateral horizontal positions, the FFA had significantly different RRV1s in eccentricity dimension (p ≤ 0.05) but PPA did not. Moreover, there was an interaction between region and eccentricity (p = 0.03). We considered that the compensation mechanisms for the peripheral field may be in FFA but not in PPA. The upper vertical positions had a main effect of eccentricity but the lower vertical positions did not. Furthermore, there was no interaction between region and eccentricity (p ≥ 0.35) at the upper and lower vertical positions, and we speculated that the difference in compensation mechanisms between FFA and PPA became weaker. The models of RRV1s relative to eccentricity and meridian factors were associated with region in the ventral category-selective areas.

### The Influence of Perceptual Experiences on Human Visual Cortex

Processing consistent visual information from V1, FFA and PPA areas manifested different processing strategies in terms of eccentricity, meridian and region, which might imply compensation mechanisms for the peripheral field. These findings did not clearly support the resolution-need hypothesis [15], [23]. An alternative explanation for the different versions of the neural activity models for the face- and house-selective areas might appeal, instead, to the statistics based on experience. Through this experience hypothesis, the compensation mechanism for the peripheral field on the higher visual cortex may be comprehended [38].

As mentioned above, the different processing strategies for the eccentricity and meridian dimensions in the human visual cortex were adopted to compensate for the non-uniformity of the human retina and V1 [47], [48], [58], which resulted in weaker perceptual abilities in the peripheral field and the upper vertical positions [38], [54], [55]. We propose that the compensation mechanism is driven by visual perception needs. Moreover, specific modes for each category of stimuli correspond to the retinal location in which those objects are typically observed. Generally, the perception of face images is optimal with high-resolution foveal information [15], [23], [38]. However, faces may appear in the entire visual field. Thus, FFA exhibited RRV1s greater than 1.33 (Figure 6, 9), and the increasing RRV1s appear to have a role as a compensation mechanism for the peripheral faces. In contrast, house and sense perceptions provide more relevant visual information from the periphery in the everyday perceptual experience. Thus, PPA correspondingly exhibit smaller RRV1s (Figure 6, 9) and weaker or no compensation mechanisms for the peripheral houses. These associations might reflect how experience affects the locations of where these stimuli are typically observed in daily life. Moreover, natural selection may have also led to heritable differences between these areas [38].

## Conclusion

From our results, the neural response amplitudes and the values of RRCPs demonstrated the significant differences for each position in V1, FFA, and PPA. Measuring the RRV1s, we found that the FFA and PPA process the visual information from V1 using different neural processing strategies. The first was the dimension of eccentricity, which the values of RRV1s at the central positions were smaller than those at the peripheral positions in FFA at the contralateral horizontal positions and upper vertical positions, and in PPA only at the upper vertical positions. The second was the dimension of meridian, which the RRV1s observed at the upper vertical positions were greater than those at the lower vertical positions. The third was the dimension of region, which the RRV1s in FFA were greater than those in the PPA, and the significantly increasing trends of RRV1s were observed in FFA. The findings reported here suggested that the ventral category-selective areas develop specific modes to process stimuli located at different positions, depending on the retinal locations of where the object is typically observed in daily life. Taken together, these different neural processing strategies of the ventral visual cortex might be shaped by experience.

## Supporting Information

Figure S1 **Neural Response to the images of faces and houses in bilateral V1, FFA and PPA.**
(DOC)

Table S1 **The mean Talairach coordinates, the cluster volumes, and the defined numbers of ROI in V1 for faces and houses at each position.**
(DOC)

Table S2 **Mean behavioral performance during object experiments.**
(DOC)

File S1 **Statistical values lists of linear mixed models for repeated measures.**
(DOC)
