# Supplementary material for: Regional Neural Response Differences in the Determination of Faces or Houses Positioned in a Wide Visual Field
Source: PLoS One. 2013 Aug 21;8(8):e72728. doi: 10.1371/journal.pone.0072728 (PMC3749153; doi:10.1371/journal.pone.0072728)
Supplement: Figure S1 — Neural Response to the images of faces and houses in bilateral V1, FFA and PPA. (DOC) [file pone.0072728.s001.doc]

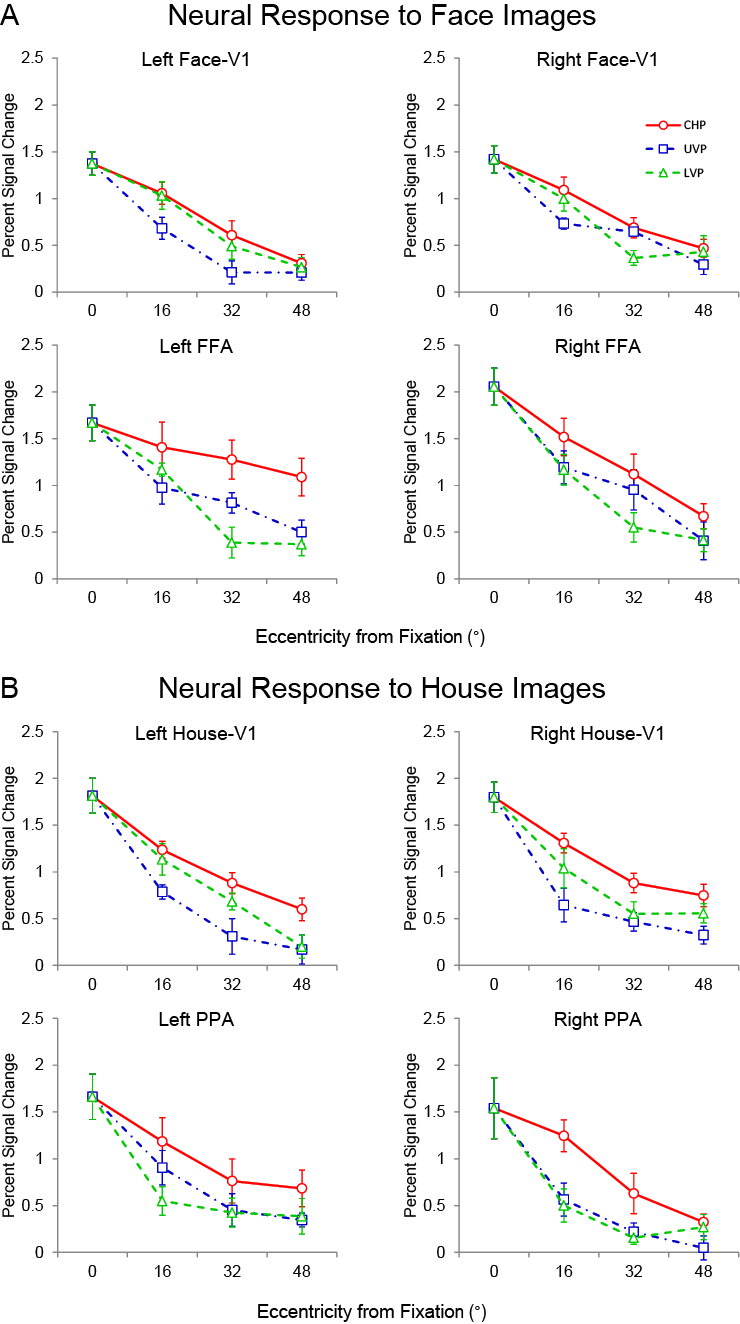


Figure S1. Neural Response to the images of faces and houses in bilateral V1, FFA and PPA. (A) Neural Response to the face images in V1 and FFA. (B) Neural Response to the house images in V1 and PPA.
