# Supplementary material for: Regional Neural Response Differences in the Determination of Faces or Houses Positioned in a Wide Visual Field
Source: PLoS One. 2013 Aug 21;8(8):e72728. doi: 10.1371/journal.pone.0072728 (PMC3749153; doi:10.1371/journal.pone.0072728)
Supplement: Table S2 — Mean behavioral performance during object experiments. (DOC) [file pone.0072728.s003.doc]

Table S2. Mean behavioral performance during object experiments.

Values are represented as the means ± SEM.
