# Supplementary material for: Regional Neural Response Differences in the Determination of Faces or Houses Positioned in a Wide Visual Field
Source: PLoS One. 2013 Aug 21;8(8):e72728. doi: 10.1371/journal.pone.0072728 (PMC3749153; doi:10.1371/journal.pone.0072728)
Supplement: File S1 — Statistical values lists of linear mixed models for repeated measures. (DOC) [file pone.0072728.s004.doc]

Table A. Linear mixed models for repeated measures with factors of eccentricity (0º, 16º, 32º and 48º) and category (faces and houses, 4 × 2) for the behavior performances.

dfn: degrees of freedom numerator, dfd: degrees of freedom denominator.

Table B. A linear mixed model for repeated measures with factors of eccentricity (0º, 16º, 32º and 48º), meridian (left, right, upper, lower), and category (face and house 3 × 4 × 2) for the behavior performances.

Table C. Linear mixed models for repeated measures with factors of eccentricity (0º, 16º, 32º and 48º) and region (for faces and houses, 4 × 2) for the neural responses in V1 and ventral category-selective areas (FFA and PPA).

Table D. A linear mixed model for repeated measures with factors of eccentricity (16º, 32º and 48º), meridian (contralateral horizontal, upper vertical, and lower vertical positions), and region (3 × 3 × 2) for the neural responses in V1 and ventral category-selective areas (FFA and PPA).

Table E. Linear mixed models for repeated measures with factors of eccentricity and region (4 × 2) for the RRCPs in V1 and ventral category-selective areas (FFA and PPA).

Table F. A linear mixed model for repeated measures with factors of eccentricity, meridian, and region (3 × 3 × 2) for the RRCPs in V1 and ventral category-selective areas (FFA and PPA).

Table G. Linear mixed models for repeated measures with factors of eccentricity and region (4 × 2) for the RRV1s.

Table H. A linear mixed model for repeated measures with factors of eccentricity, meridian, and region (3 × 3 × 2) for the RRV1s.

Table I. Linear mixed models for repeated measures with factors of eccentricity and region (4 × 2) for the neural responses to the images of faces or houses, and checkerboards.

Table J. Linear mixed models for repeated measures with factors of eccentricity and region (4 × 2) for the signal intensity values in V1.

Table K. A linear mixed model for repeated measures with factors of eccentricity, meridian, and region (3 × 3 × 2) for the signal intensity values in V1.
